# Wing morphology predicts geographic range size in vespertilionid bats

**DOI:** 10.1038/s41598-019-41125-0

**Published:** 2019-03-14

**Authors:** Bo Luo, Sharlene E. Santana, Yulan Pang, Man Wang, Yanhong Xiao, Jiang Feng

**Affiliations:** 1 Key Laboratory of Southwest China Wildlife Resources Conservation of Ministry of Education, China West Normal University, 1 Shida Road, Nanchong, 637002 China; 2 Jilin Provincial Key Laboratory of Animal Resource Conservation and Utilization, Northeast Normal University, 2555 Jingyue Street, Changchun, 130117 China; 3 Department of Biology and Burke Museum of Natural History and Culture, University of Washington, Seattle, WA 98195 USA; 4 College of Animal Science and Technology, Jilin Agricultural University, Changchun, 130117 China

## Abstract

Why some species are widespread across continents while others are confined geographically remains an open question in ecology and biogeography. Previous research has attempted to explain interspecific variation in geographic range size based on differences in dispersal ability. However, the relationship between dispersal ability and geographic range size remains uncertain, particularly in mammals. The goal of this study is to test whether geographic range size can be predicted by dispersal capacity among vespertilionid bats within a phylogenetic comparative framework. We integrated a large dataset on range area, longitudinal extent, wing morphology (a proxy for dispersal ability), migratory habit, and biogeographic realm across 126 vespertilionid bat species. We used phylogenetic regressions to disentangle the associations between these predictor factors and species range size while controlling for the effects of migration and biogeographic realm. Our analyses revealed that bat species with higher wing loading exhibit larger distribution ranges than those with lower wing loading, and that the size of geographic ranges was associated with wing aspect ratio in bats. These results highlight the relationship between wing morphology and range size in flying mammals, and suggest a role of dispersal capacity in shaping species’ geographic distributions.

## Introduction

Why some species are widespread across continents while others are confined geographically remains obscure, despite the sustained interest from ecologists and evolutionary biologists^1–6^. Geographic range size—the extent of a species’ occurrence—is a basic biogeographic variable used to determine population abundance and survival^7^. As a consequence, geographic range size has been an important parameter used to assign species’ extinction risk in the Red List of the International Union for Conservation of Nature^8^. A deeper understanding of the causes of interspecific variation in geographic range size has important implications for biodiversity conservation, in particular under global climate change scenarios^9,10^.

A multitude of ecological and evolutionary processes may constrain species’ geographic distributions. One of the most common explanations for interspecific differences in geographic range size is the site colonization hypothesis, which emphasizes the importance of dispersal ability^1,7,11^. Because long-distance dispersal confers the ability to colonize new habitats, species with greater dispersal ability are expected to have larger distribution ranges compared with poor dispersers^11–13^. Support for the site colonization hypothesis has been found in some terrestrial and aquatic organisms. For example, ecomorphological predictors of dispersal distance account for the observed variation in range area among warblers^14^. In Indo-Pacific coral reef fishes, species’ range areas are predicted by the duration of pelagic larval phrase, a good surrogate for dispersal potential^13^. In freshwater insects, mayflies with high dispersal potential tend to be more widespread than poorly dispersing species^15^. Nonetheless, it remains unknown if dispersal ability affects the sizes of geographic ranges in most mammal groups.

Bats are one of the most species-rich group of mammals, representing over 20% of all extant mammal species^16^. They are distributed globally, except in the polar regions and some isolated islands, presumably owing to their ability for powered flight^17^. It has been shown that bat wing morphology determines their ability to disperse into new geographic regions^18,19^. In general, bats with higher wing loading (i.e., larger body mass relative to wing surface area) fly at higher speeds and greater distances^18^, allowing them to extend their ranges even in fragmented landscapes. In addition, bats with higher aspect ratio (i.e., greater wing length relative to wing width) suffer lower drag and enhanced aerodynamic efficiency during flight^20^, which may facilitate long-distance movement across geographic barriers and ultimately range expansion. Prior studies have focused on the effects of ecological factors on biogeographic patterns among some bat lineages^21–23^. It is still unclear, however, whether the site colonization hypothesis holds for bats, especially in a phylogenetic context.

Here, we aim to assess the influence of dispersal ability on geographic range size in vespertilionid bats through phylogenetic comparative analyses. Vespertilionidae is the largest family of bats, and has successfully colonized all the biogeographic regions in the world^24^. Most vespertilionids are insectivores, albeit some *Myotis* and *Nyctalus* species also capture small fishes or passerine birds^25^. These bats show marked diversity in wing morphology, resulting in remarkable differences in flight performance among species^18,19,26^. To test the site colonization hypothesis, we compiled a dataset spanning 126 vespertilionid bat species, which included range area, longitudinal extent, wing morphology, migratory habit, and biogeographic realm. We quantified range area and longitudinal extent for each species using distribution maps from the IUCN Red List^17^. Following previous research^27^, we used relative wing loading and aspect ratio as indirect measures of dispersal ability. We examined the relationship between wing morphology and species range size, while accounting for the effects of migratory habit and biogeographic realm. Since IUCN range polygons encompass the known species occurrences based on published literature and field surveys^17^, and this may underestimate the actual geographic distributions among rare and poorly studied species, we repeated our analyses excluding species with range areas below the 10th percentile. Given that bat wing morphology is associated with foraging ecology^18^, we also conducted additional analyses while controlling for foraging guild. If dispersal ability is a significant determinant of geographic range sizes in bats, we expected that: (1) relative wing loading would be positively related to range area and longitudinal extent; and (2) species with greater aspect ratios would be more geographically widespread relative to those with lower aspect ratios.

## Results

**Figure 1:**
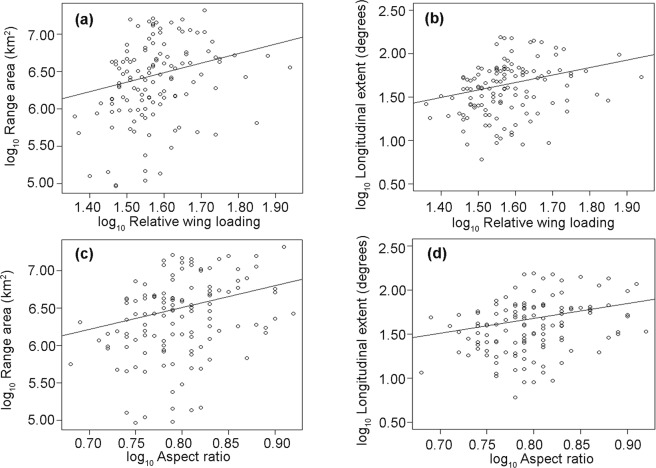
Relationship between dispersal ability and geographic range size in vespertilionid bats. The scatterplots depict the relationship between (**a**) log_10_ relative wing loading and log_10_ range area, (**b**) log_10_ relative wing loading and log_10_ longitudinal extent, (**c**) log_10_ aspect ratio and log_10_ range area, and (**d**) log_10_ aspect ratio and log_10_ longitudinal extent. Lines represent the best-fitting regression models after correcting for phylogeny, migration, and biogeographic realm.

There was considerable interspecific variation in geographic range sizes among vespertilionid bats. The smallest range area in our data set was 9.29 e + 4 km^2^, whereas the largest range area reached 2.10 e + 7 km^2^. The longitudinal extent of species ranged from 6.08 to 156.30 degrees. A lambda model, which converts the phylogeny into a covariance matrix with an error term, provided the best fit when testing for the relationship among wing morphology, range area, and longitudinal extent (Table 1). After controlling for the effects of migration and biogeographic realm, relative wing loading was positively associated with range area (N = 126, *R*^2^ = 0.069, estimate ± s. e. = 1.27 ± 0.51, *t* = 2.49, *P* = 0.014; Fig. 1a) and longitudinal extent (N = 126, *R*^2^ = 0.097, estimate ± s. e. = 0.86 ± 0.28, *t* = 2.99, *P* = 0.0033; Fig. 1b). A significant positive correlation was observed between aspect ratio and species range size (range area: N = 126, *R*^*2*^ = 0.074, estimate ± s. e. = 2.91 ± 0.99, *t* = 2.92, *P* = 0.0042; longitudinal extent: N = 126, *R*^*2*^ = 0.083, estimate ± s. e. = 1.69 ± 0.57, *t* = 2.95, *P* = 0.0037; Fig. 1c,d). Additional analyses using different sample sizes yielded similar results (Tables S1-S3; Figs S1-S3).

**Table 1:** Summary of regression models under different evolutionary scenarios. In each model, range area or longitudinal extent were predicted by relative wing loading (RWL) and aspect ratio (AR).

Range size	Factor	Model	AICc	*R* ^*2*^	Estimate	*P*-value
Range area	RWL	BM	269.50	0.080	0.40 ± 0.63	0.52
		OU	211.30	0.10	1.64 ± 0.46	0.0005
		**λ**	**209.30**	**0.069**	**1.27** ± **0.51**	**0.014**
		OLS	209.40	0.11	1.69 ± 0.45	0.0003
	AR	BM	268.90	0.077	0.45 ± 0.96	0.64
		OU	209.80	0.11	3.69 ± 0.99	0.0003
		**λ**	**205.50**	**0.074**	**2.91** ± **0.99**	**0.0042**
		OLS	208.10	0.11	3.73 ± 0.99	0.0002
Longitudinal extent	RWL	BM	119.60	0.096	0.32 ± 0.34	0.35
		OU	71.80	0.098	0.87 ± 0.26	0.001
		**λ**	**70.80**	**0.097**	**0.86** ± **0.28**	**0.0033**
		OLS	72.50	0.098	0.88 ± 0.25	0.0009
	AR	BM	118.40	0.084	0.58 ± 0.52	0.26
		OU	70.40	0.098	1.89 ± 0.56	0.001
		**λ**	**69.40**	**0.083**	**1.69** ± **0.57**	**0.0037**
		OLS	72.50	0.098	0.87 ± 0.25	0.0009

The models tested were: Brownian motion (BM), Ornstein-Uhlenbeck (OU), lambda (λ), and ordinary least square regression (OLS). Estimate denotes the coefficient of regression. The best-fitting models are noted in bold.

## Discussion

The site colonization hypothesis predicts that a species’ geographic range size is shaped by its ability to disperse from one habitat to another^7,11,12^. This is the case in a broad range of organisms, including plants^28^, insects^15,29^, mollusks^30^, fishes^13^, amphibians^31^, and birds^14,32^. However, the relationship between dispersal ability and geographic range size is uncertain in most mammal groups. Here, we test dispersal-based hypothesis by applying a phylogenetic framework across a large sample of bat species at a global scale. Our comparative analyses revealed that the geographic range sizes of bats are positively associated with wing loading and aspect ratio, two commonly used indicators of dispersal ability. Bats with higher wing loading and greater aspect ratios tend to be more geographically widespread. These results provide the first evidence, to our knowledge, that dispersal ability plays a role in shaping geographic range size in flying mammals.

The wing morphology of bats contributes significantly to interspecific variation in geographic range size, even after accounting for the effects of phylogeny, migration and geographic location. This is consistent with previous research on mayflies and stoneflies, which have shown a positive link between forewing length and the geographic areas occupied by species^15,33^. Similarly, wing length and shape explain a large proportion of the interspecific variation in range area among warblers^14^. In Himalayan birds, the maximum northern latitude of species’ geographic ranges is affected by the hand-wing index, which reflects aspect ratio on a size-independent scale^34^. These results indicate that high wing loading and aspect ratio provide an opportunity for dispersal over small and moderate natural barriers, enabling species to colonize diverse geographic regions across fragmented landscapes^1,7,11^. A positive feedback between species’ range expansions and evolution of wing morphology, as observed in butterfly^35^ and bush crickets^36^, could further accelerate range shifts. Consequently, wing morphology seems to act as an important factor shaping species range size in bats and other flying animals.

Biological dispersal differs from migration. While the former refers to random movement of individuals from one site to another, the latter comprises seasonal round-trip movement by all or part of a population^37^. Therefore, dispersal distance is not equivalent to migration distance, although they are related to each other^38^. Bats are capable of powered flight, allowing them to achieve long-distance dispersal over oceans, deserts, and mountains^26,39^. However, directly quantifying realized dispersal distances in bats remains a great challenge. Identifying the traits associated with dispersal distance would be valuable for predicting the dispersal ability of bats^27^. It has been demonstrated that wing loading influences the rate of gene flow between populations in several bat families, such as Vespertilionidae, Rhinolophidae, and Molossidae^27,40^. There is also a positive association between aspect ratio and maximum movement distance in some European bats^19^. These findings echo previous studies in other taxa^41,42^, and suggest that wing morphology can serve as a reliable indicator of dispersal ability in bats.

Bats are important bio-indicators for global climate change^43^. It is still unclear whether most bats will encounter range loss and rapid decline in numbers as climate envelope models have predicted^23^. Field observations suggest that warming climates are causing some bat species to expand their ranges towards northern latitudes and higher elevations, e.g., great stripe-faced bats (*Vampyrodes caraccioli*)^44^, common vampire bats (*Desmodus rotundus*)^44^, Kuhl’s pipistrelle (*Pipistrellus kuhlii*)^45^, and Nathusius’ pipistrelle (*Pipistrellus nathusii*)^46^. However, some bats may remain in their current habitats due to lower dispersal ability and niche specialization^22^. Both of these scenarios involve high risks of extinction. *In situ*, altered climates could reach or surpass the upper limits of thermal tolerance, gradually causing population declines and extinction^47^. Additionally, range shifts in bats may lag behind climate change as a result of limited dispersal and multispecies interaction in the new biomes^3,48^. Long-distance dispersal through human-dominated landscapes also has negative impacts on population connectivity and reproductive success^49^. Further research is critically needed to explore whether and how populations at the margins of bat distributions respond to climate change via dispersal.

In summary, we employ a phylogenetic comparative approach to elucidate the relationship between dispersal ability and geographic range size in vespertilionid bats. By incorporating migratory status and biogeographic realm, we demonstrate that wing loading and aspect ratio are significant, positive predictors of species range size, suggesting that dispersal ability is important in shaping the geographic ranges of vespertilionid bats. However, dispersal ability is not the sole determinant of range size in bats, given that wing parameters account for only about 7–10% of the variance in range size. Life history traits, ecological niche breadth, and anthropogenic pressures may also affect a species’ successful establishment following dispersal, and thus geographic range size^50^. Investigating these factors was beyond the scope of this study. Given the robust association between species range size and extinction risk^51,52^, our findings highlight that wing morphology may inform conservation priorities for bat species. Coupled with previous comparative studies^4,50^, our findings help understand the factors that contribute to the great diversity of geographic range sizes across mammals.

## Materials and Methods

### Data Collection

A total of 126 bat species from the family Vespertilionidae (Supplementary dataset) were selected for this study based on three criteria: (1) distribution maps could be obtained from the IUCN Red List^17^; (2) detailed phylogenetic information was available on the most recent mammal supertree^53^; and (3) information on body mass, wing loading, and aspect ratio could be collected. The average value of each morphological trait was used if data differed among published sources. *Corynorhinus townsendii*, *C*. *rafinesquii*, *Neoromicia capensis*, *N*. *zuluensis*, *N*. *tenuipinnis*, *N*. *nana*, *Vespadelus regulus*, *V*. *vulturnus*, *V*. *pumilus*, *Pipistrellus cadornae*, *Pipistrellus pulveratus*, *Nycticeinops schlieffeni*, and *Glauconycteris variegata* were regarded as synonyms of *Plecotus townsendii*, *P*. *rafinesquii*, *Eptesicus capensis*, *E*. *zuluensis*, *E*. *tenuipinnis*, *Pipistrellus nanus*, *E*. *regulus*, *E*. *vulturnus*, *E*. *pumilus*, *Hypsugo cadornae*, *H*. *pulveratus*, *Nycticeius Schlieffeni*, and *Chalinolobus variegatus*^17^, respectively.

We extracted species range polygons from the IUCN database^17^. We calculated the total area (km^2^) and longitudinal extent (degrees) of polygons to quantify species range size using cylindrical equal area projections^50^. Each species was assigned to the biogeographic realm covering more than 80% of its distribution range. These biogeographic realms were: Palearctic, Sino-Japanese, Oriental, Australian, Oceanic, Afrotropical, Neotropical, and Nearctic^24^. Species with less than 80% of their distribution range in any given realm were classified as cosmopolitan.

To quantify dispersal ability per species, we compiled data on wing loading, aspect ratio, and body mass from the literature (Supplementary dataset). We used relative wing loading (RWL = WL/body mass^1/3^) in our analyses to correct for allometric effects^54^. We defined migration as seasonal movements from one region to another. Seasonal shifts in roost use greater than 100 km were also regarded as migratory movements^26^. We defined migratory habits as non-migration (<100 km), short-distance migration (ranged from 100 to 1,000 km), long-distance migration (>1,000 km), and uncertain status^26^. We log10-transformed the values of range size and wing morphology to achieve a normal distribution.

### Statistical Analyses

We tested for associations between predictor factors and species range size through phylogenetic generalized least square (PGLS) regressions based on a pruned supertree^53^. Bat range size was entered into the regression models as a dependent variable. Relative wing loading and aspect ratio were treated as fixed predictor variables. Migratory habit and biogeographic realm of species were assigned as covariates. We ran PGLS regressions based on four different evolutionary models (Brownian motion, Ornstein-Uhlenbeck, lambda, and ordinary least square) using the packages nlme^55^ and MuMIn^56^. The Brownian motion model assumes that the traits change gradually through time with a constant rate, the Ornstein-Uhlenbeck model fits a random walk with a deterministic tendency for trait values, and the lambda model represents a modification of the Brownian motion model after correcting for the phylogenetic covariance matrix. We chose the best regression model according to the Akaike information criterion corrected for small sample size (AICc)^57^. To verify the robustness of our results, we re-analyzed the data while excluding migratory species and those with range areas below the 10th percentile. We also performed additional analyses while incorporating information about foraging guild. All statistics were performed in R 3.3.3.

## Supplementary information

Supplementary information

Supplementary dataset

## Data Availability

The datasets generated and/or analyzed during the current study are presented in the supplementary material.
