# Supplementary material for: Wing morphology predicts geographic range size in vespertilionid bats
Source: Sci Rep. 2019 Mar 14;9:4526. doi: 10.1038/s41598-019-41125-0 (PMC6418303; doi:10.1038/s41598-019-41125-0)
Supplement: Supplementary file 1 — Supplementary information [file 41598_2019_41125_MOESM1_ESM.docx]

**Supplementary Information**

**Wing morphology** **predicts geographic range size in vespertilionid bats**

Bo Luo^1, 2,*^, Sharlene E. Santana^3^, Yulan Pang^1^, Man Wang^1^, Yanhong Xiao^2^, and Jiang Feng^2, 4,*^

| **Range size** | **Factor** | **Model** | **AICc** | ***R^2^*** | **Estimate** | ***P*-value** |
| --- | --- | --- | --- | --- | --- | --- |
| Range area | RWL | BM | 191.70 | 0.018 | 0.28 ± 0.76 | 0.71 |
|  |  | **OU** | **136.00** | **0.080** | **1.32 ± 0.51** | **0.011** |
|  |  | λ | 136.80 | 0.033 | 0.84 ± 0.56 | 0.13 |
|  |  | OLS | 136.10 | 0.083 | 1.35 ± 0.51 | 0.010 |
|  | AR | BM | 191.00 | 0.017 | 0.18 ± 1.14 | 0.87 |
|  |  | OU | 137.20 | 0.071 | 2.98 ± 1.24 | 0.018 |
|  |  | **λ** | **133.70** | **0.042** | **2.30 ± 1.23** | **0.064** |
|  |  | OLS | 135.00 | 0.076 | 3.09 ± 1.24 | 0.014 |
| Longitudinal extent | RWL | BM | 72.00 | 0.092 | 0.24 ± 0.38 | 0.51 |
|  |  | **OU** | **29.40** | **0.086** | **0.64** ± 0.28 | **0.025** |
|  |  | λ | 31.80 | 0.086 | 0.68 ± 0.30 | 0.026 |
|  |  | OLS | 30.30 | 0.087 | 0.70 ± 0.27 | 0.013 |
|  | AR | BM | 70.90 | 0.080 | 0.45 ± 0.57 | 0.43 |
|  |  | **OU** | **29.40** | **0.070** | **1.45 ± 0.67** | **0.033** |
|  |  | λ | 30.90 | 0.069 | 1.38 ± 0.67 | 0.044 |
|  |  | OLS | 29.90 | 0.072 | 1.49 ± 0.67 | 0.029 |

**Table S1.** Summary of regression models for non-migratory bats (N = 89). In each model, range area or longitudinal extent were predicted by relative wing loading (RWL) and aspect ratio (AR). The models tested were: Brownian motion (BM), Ornstein-Uhlenbeck (OU), lambda (λ), and ordinary least square regression (OLS). Estimate denotes the coefficient of regression. The best-fitting models are noted in bold.


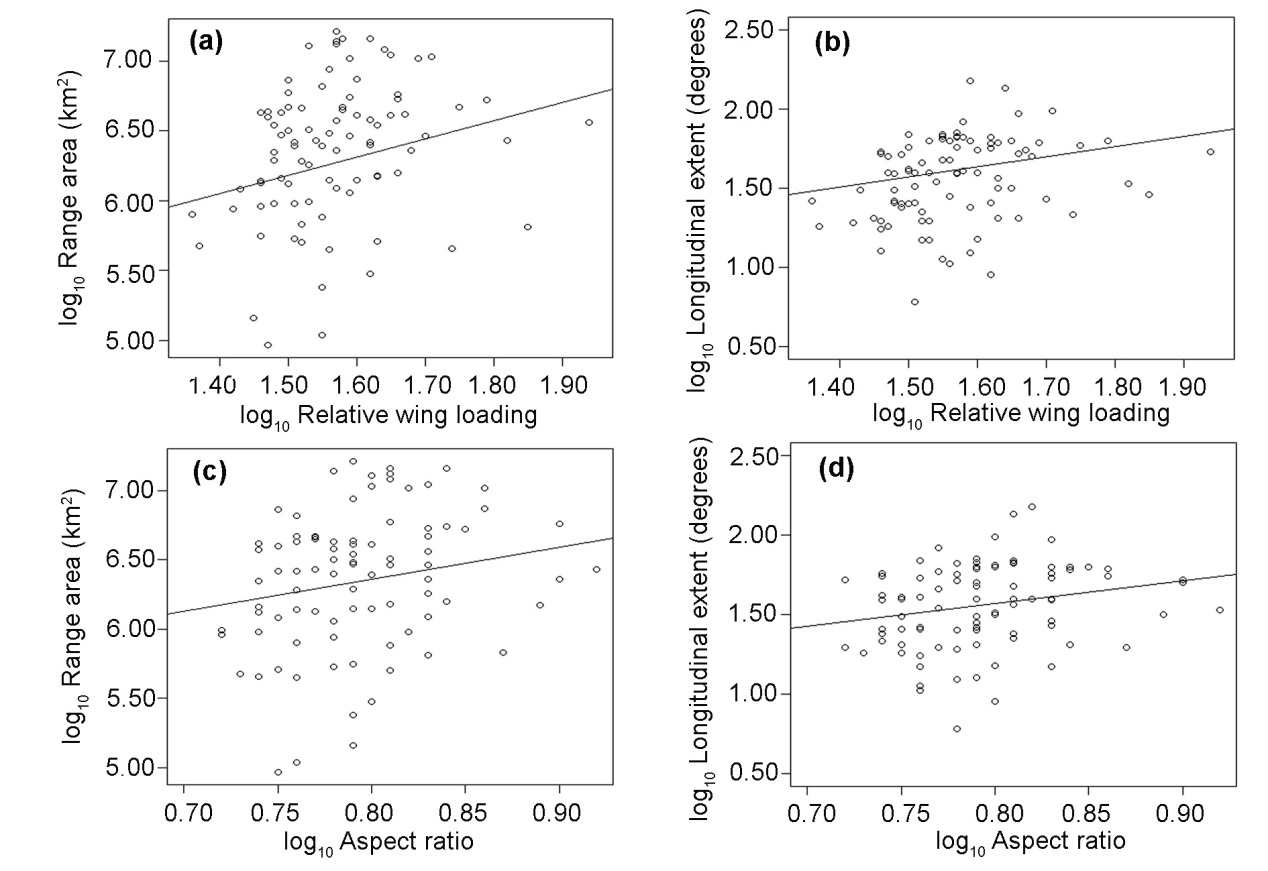


**Figure S1.** Relationship between wing morphology and range size in non-migratory bats (N = 89). The scatterplots depict the relationship between **(a)** log_10_ relative wing loading and log_10_ range area, **(b)** log_10_ relative wing loading and log_10_ longitudinal extent, **(c)** log_10_ aspect ratio and log_10_ range area, and **(d)** log_10_ aspect ratio and log_10_ longitudinal extent. Lines represent the best-fitting regression models after correcting for phylogeny and biogeographic realm.

| **Range size** | **Factor** | **Model** | **AICc** | ***R^2^*** | **Estimate** | ***P*-value** |
| --- | --- | --- | --- | --- | --- | --- |
| Range area | RWL | BM | 181.20 | 0.086 | 0.56 ± 0.47 | 0.24 |
|  |  | **OU** | **131.70** | **0.11** | **1.31 ± 0.37** | **0.0006** |
|  |  | λ | 132.40 | 0.094 | 1.18 ± 0.40 | 0.00379 |
|  |  | OLS | 132.00 | 0.11 | 1.30 ± 0.37 | 0.0006 |
|  | AR | BM | 179.60 | 0.081 | 1.05 ± 0.72 | 0.15 |
|  |  | OU | 133.00 | 0.11 | 2.68 ± 0.79 | 0.0009 |
|  |  | **λ** | **130.40** | **0.086** | **2.38 ± 0.79** | **0.0032** |
|  |  | OLS | 131.00 | 0.11 | 2.73 ± 0.79 | 0.0008 |
| Longitudinal extent | RWL | BM | 99.10 | 0.048 | 0.47 ± 0.32 | 0.15 |
|  |  | **OU** | **42.90** | **0.12** | **0.90** ± 0.24 | **0.0004** |
|  |  | λ | 43.70 | 0.12 | 0.94 ± 0.26 | 0.0006 |
|  |  | OLS | 43.40 | 0.12 | 0.90 ± 0.25 | 0.0004 |
|  | AR | BM | 98.80 | 0.025 | 0.61 ± 0.50 | 0.23 |
|  |  | **OU** | **45.10** | **0.087** | **1.62 ± 0.53** | **0.0029** |
|  |  | λ | 46.20 | 0.076 | 1.53 ± 0.54 | 0.0052 |
|  |  | OLS | 45.50 | 0.088 | 1.63 ± 0.53 | 0.00298 |

**Table S2.** Summary of regression models while excluding species with range area below the 10th percentile (N = 113). In each model, range area or longitudinal extent were predicted by relative wing loading (RWL) and aspect ratio (AR). The models tested were: Brownian motion (BM), Ornstein-Uhlenbeck (OU), lambda (λ), and ordinary least square regression (OLS). Estimate denotes the coefficient of regression. The best-fitting models are noted in bold.


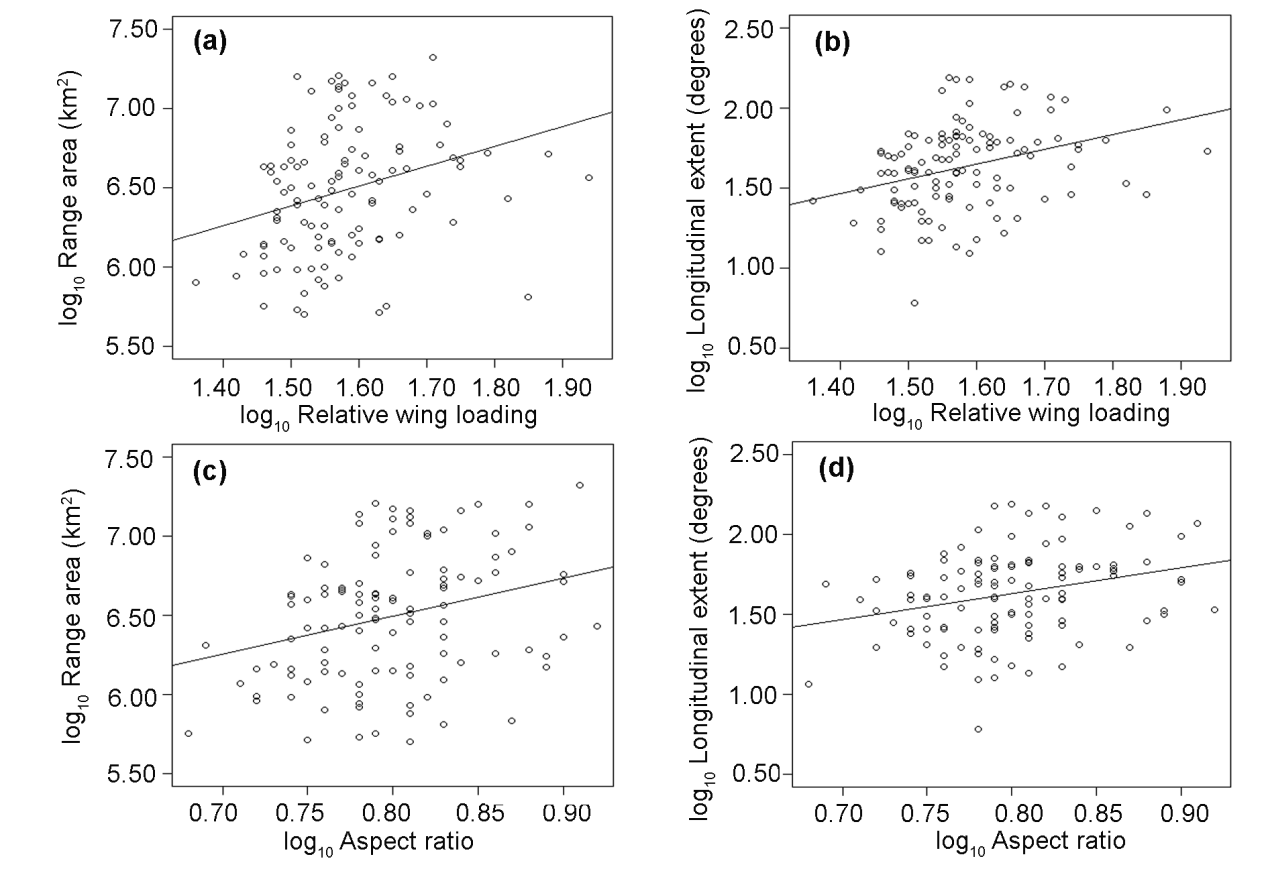


**Figure S2.** Relationship between wing morphology and range size while excluding species with range area below the 10th percentile (N = 113). The scatterplots depict the relationship between **(a)** log_10_ relative wing loading and log_10_ range area, **(b)** log_10_ relative wing loading and log_10_ longitudinal extent, **(c)** log_10_ aspect ratio and log_10_ range area, and **(d)** log_10_ aspect ratio and log_10_ longitudinal extent. Lines represent the best-fitting regression models after correcting for phylogeny, migration, and biogeographic realm.

| **Range size** | **Factor** | **Model** | **AICc** | ***R^2^*** | **Estimate** | ***P*-value** |
| --- | --- | --- | --- | --- | --- | --- |
| Range area | RWL | BM | 110.70 | 0.065 | -0.67 ± 0.64 | 0.29 |
|  |  | OU | 71.20 | 0.071 | 0.46 ± 0.52 | 0.38 |
|  |  | λ | 71.30 | 0.070 | 0.44 ± 0.52 | 0.39 |
|  |  | **OLS** | **68.90** | **0.070** | **0.45 ± 0.52** | **0.39** |
|  | AR | BM | 110.70 | 0.036 | 0.62 ± 0.86 | 0.47 |
|  |  | OU | 65.00 | 0.13 | 2.26 ± 0.91 | 0.016 |
|  |  | λ | 64.90 | 0.14 | 2.34 ± 0.92 | 0.013 |
|  |  | **OLS** | **62.60** | **0.14** | **2.26 ± 0.91** | **0.016** |
| Longitudinal extent | RWL | BM | 58.50 | 0.084 | -0.35 ± 0.43 | 0.412 |
|  |  | OU | 16.40 | 0.076 | 0.69 ± 0.34 | 0.051 |
|  |  | λ | 15.30 | 0.057 | 0.67 ± 0.32 | 0.045 |
|  |  | **OLS** | **14.10** | **0.075** | **0.68 ± 0.34** | **0.052** |
|  | AR | BM | 58.50 | 0.067 | 0.081 ± 0.58 | 0.89 |
|  |  | OU | 13.40 | 0.10 | 1.51 ± 0.62 | 0.017 |
|  |  | **λ** | **10.80** | **0.085** | **1.39 ± 0.59** | **0.022** |
|  |  | OLS | 11.10 | 0.10 | 1.52 ± 0.62 | 0.021 |

**Table S3.** Summary of regression models while controlling for the effect of foraging guild (N = 70). In each model, range area or longitudinal extent were predicted by relative wing loading (RWL) and aspect ratio (AR). The models tested were: Brownian motion (BM), Ornstein-Uhlenbeck (OU), lambda (λ), and ordinary least square regression (OLS). Estimate denotes the coefficient of regression. The best-fitting models are noted in bold.


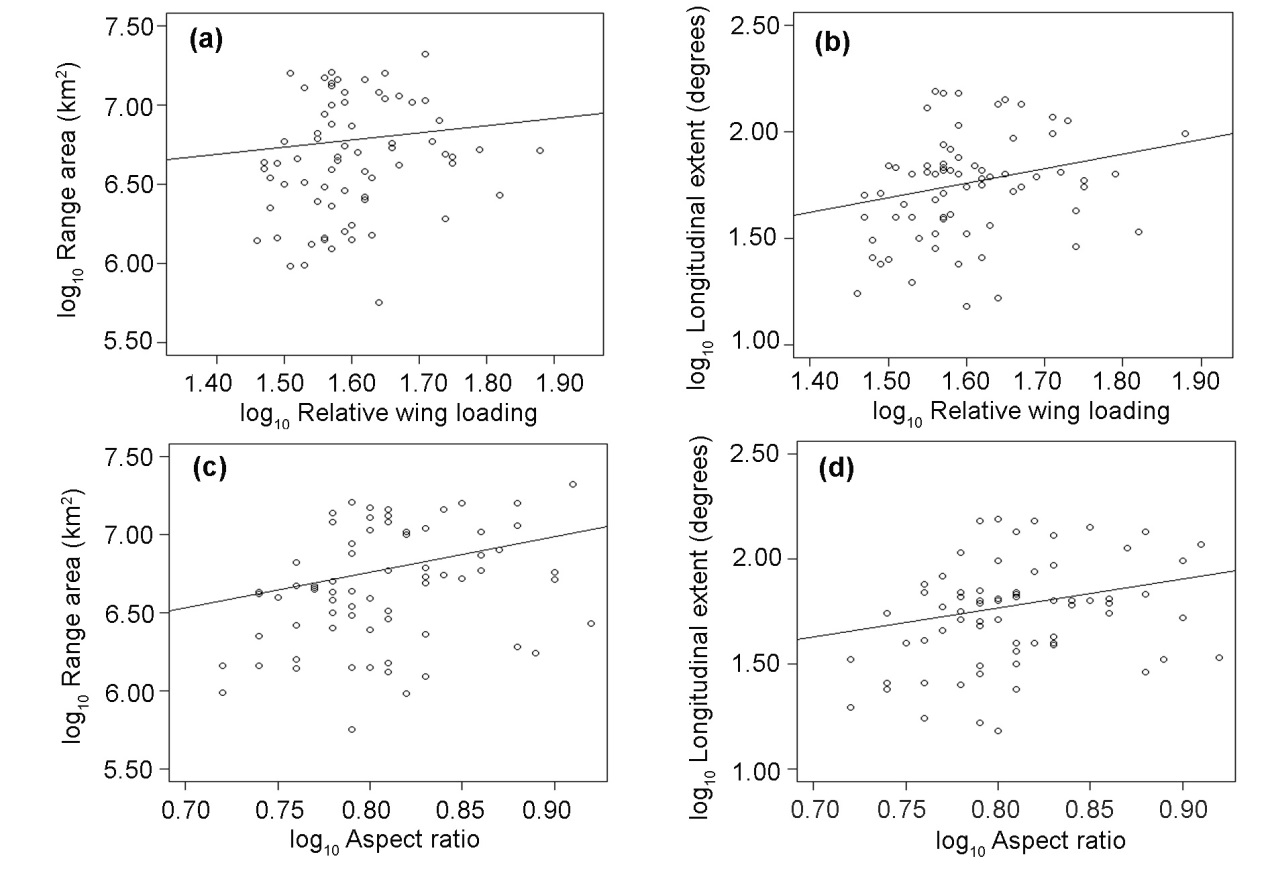


**Figure S3.** Relationship between wing morphology and range size while controlling for the effect of foraging guild (N = 70). The scatterplots depict the relationship between **(a)** log_10_ relative wing loading and log_10_ range area, **(b)** log_10_ relative wing loading and log_10_ longitudinal extent, **(c)** log_10_ aspect ratio and log_10_ range area, and **(d)** log_10_ aspect ratio and log_10_ longitudinal extent. Lines represent the best-fitting regression models after correcting for foraging guild.
